# Cancer outcomes research—a European challenge: measures of the cancer burden

**DOI:** 10.1002/1878-0261.13012

**Published:** 2021-06-22

**Authors:** Mette Kalager, Hans‐Olov Adami, Pernilla Lagergren, Karen Steindorf, Paul W. Dickman

**Affiliations:** ^1^ Clinical Effectiveness Research Group University of Oslo and Oslo University Hospital Norway; ^2^ Department of Medical Epidemiology and Biostatistics Karolinska Institutet Stockholm Sweden; ^3^ Surgical Care Science Department of Molecular Medicine and Surgery Karolinska Institutet Karolinska University Hospital Stockholm Sweden; ^4^ Department of Surgery and Cancer Imperial College London UK; ^5^ Division of Physical Activity, Prevention and Cancer National Center for Tumor Diseases (NCT) and German Cancer Research Center (DKFZ) Heidelberg Germany

**Keywords:** cancer, health‐related quality of life, incidence, mortality, outcomes, survival

## Abstract

In a mission that aims to improve cancer control throughout Europe, the European Academy of Cancer Sciences has defined two key indicators of progress: within one to two decades, overall cancer‐specific 10‐year survival should reach 75%, and in each country, overall cancer mortality rates should be convincingly declining. To lay the ground for assessment of progress and to promote cancer outcomes research in general, we have reviewed the most common population‐based measures of the cancer burden. We emphasize the complexities and complementary approaches to measure cancer survival and the novel opportunities for improved assessment of quality of life. We propose that: incidence and mortality rates are standardized to the European population; net survival is used as the measure of prognosis but with proper adjustments for confounding when temporal trends in overall cancer survival are assessed; and cancer‐specific quality of life is measured by a combination of existing questionnaires and utilizes emerging communication technologies. We conclude that all measures are important and that a meaningful interpretation also requires a deep understanding of the larger clinical and public health context.

## Graphical abstract

This review defines the many measures used to quantify the burden of cancer, their pros, cons, and utility for different purposes. Recommendations are provided for the most informative measures and the methodologic challenges that need accommodation in assessing the Academy's goals. The needs and opportunities for development of and standardization of cancer‐specific quality of life assessments is also emphasized.

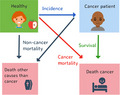

**Abbreviations**
EORTC QLQ: Quality of Life Questionnaire‐Core 30
EORTC: European Organization for Research and Treatment of Cancer
FACT‐G: Functional Assessment of Cancer Therapy‐General
QALY: quality‐adjusted life‐year

## 1 Introduction

In 2009, the European Academy of Cancer Sciences (hereafter referred to as the Academy) was founded as a complement to existing professional organizations covering different aspects of cancer research and management in Europe. The overarching goal and raison d'être for the Academy is to strengthen cancer control throughout Europe, reduce cancer mortality, improve quality of life among millions of cancer survivors, and reduce disparities in cancer research and management between European countries. To fulfill its mission as an independent body, the Academy is supposed to provide high‐level advice to politicians and other actors in the public health arena.

After a decade of consolidation and early initiatives in some areas [1, 2, 3, 4], the Academy is now taking a quantum leap. The goals are to review extensively, throughout Europe, the entire cancer continuum from basic research to palliative care; to improve infrastructures and facilitate large‐scale collaborations; to accelerate translation from basic research to clinical implementation; to uncover disparities at all levels; and to ultimately improve prognosis and quality of life among cancer survivors.

As part of this new initiative, the Academy has charged us with the mission of investigating opportunities, challenges, and needs in outcomes research. We are citing the Academy's goals as a basis for discussing cancer control measures, but the authors did not contribute to formulating these goals and our citing them does not imply endorsement. The first part of our analysis is strongly motivated by the Academy's goal to achieve an overall 75% 10‐year cancer‐specific survival and convincing evidence of reductions in overall cancer mortality throughout Europe within the next one or two decades. Because no single calculus can capture the complex cancer burden, a necessary prelude is to discuss pros and cons with different measures of incidence, survival, mortality, and quality of life. Our message is not only that we need them all, but also that their interplay is extraordinarily complex. Further, the assessment of whether the seemingly straightforward goals of the Academy have been achieved requires deep familiarity with features of both the measures themselves and the larger clinical and public health context. The biggest challenge may indeed be to make the different measures comparable across European populations, which requires, for example, standardization of data collection, definition of malignant neoplasms, completeness of reporting of incident cancers, and use of a uniform definition of defining date of diagnosis.

Our focus is on population‐based measures of cancer burden, which can be estimated and reported for a large number of countries. These measures differ from the metrics used in clinical practice (such as caseload, case fatality rate, and relapse‐free, progression‐free, and overall survival), as data are rarely available at a population level to estimate these measures (relapse‐free and progression‐free survival) or are not suitable for conditions such as cancer with a long duration, where competing risks are an issue (case fatality rate).

## 2 Population‐based measures of cancer burden

### 2.1 Overview

**Fig. 1:**
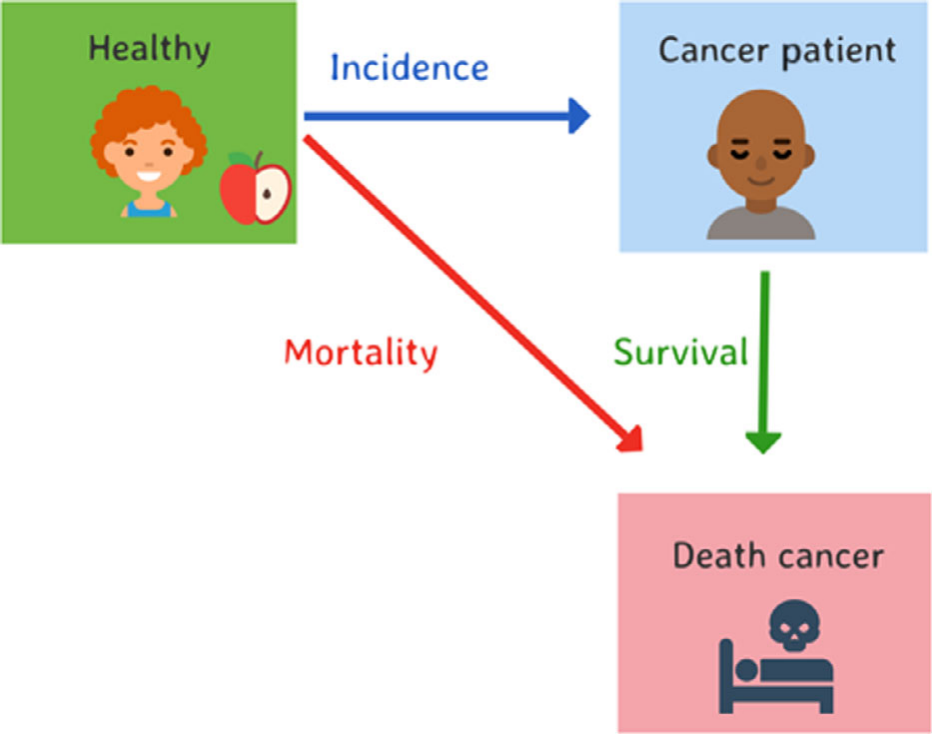
Conceptual overview of the measures that describe the rate of transitioning from one health state to another. Competing risks are disregarded.

The concepts of incidence, mortality, and survival represent the rate of transition from one health state to another. Figure 1 is a simplification of reality but serves to introduce the central concepts. Incidence represents the rate at which individuals in the population are diagnosed with cancer. It is driven by changes in the prevalence of (or exposure to) risk factors along with the intensity and accuracy of diagnostic methods (including screening). Survival represents the rate at which patients with a cancer diagnosis die of that cancer. Survival is influenced by treatment effectiveness, but also the incidence. Because survival, in this context, is estimated based on cancer patients, changes in the characteristics of the patients and their tumors will affect survival. For example, improved diagnostic techniques may result in a shift toward detection at an earlier stage and of more indolent tumors and therefore improved survival [5].

It is evident that to die of cancer, one must first develop cancer. We have drawn the arrow for mortality from ‘healthy’ to ‘dead’ in Fig. 1 to illustrate that mortality measures the rate of death due to cancer in the entire population, but we acknowledge that to die of cancer one must progress from ‘healthy’ to ‘cancer’ to ‘death’. The denominators for incidence and mortality are the entire population, whereas the denominator for survival is only patients with a cancer diagnosis. Mortality is affected by both factors that impact incidence and factors that impact survival. As such, evaluating progress against cancer requires the simultaneous interpretation of trends in incidence, mortality, and survival [6].

**Fig. 2:**
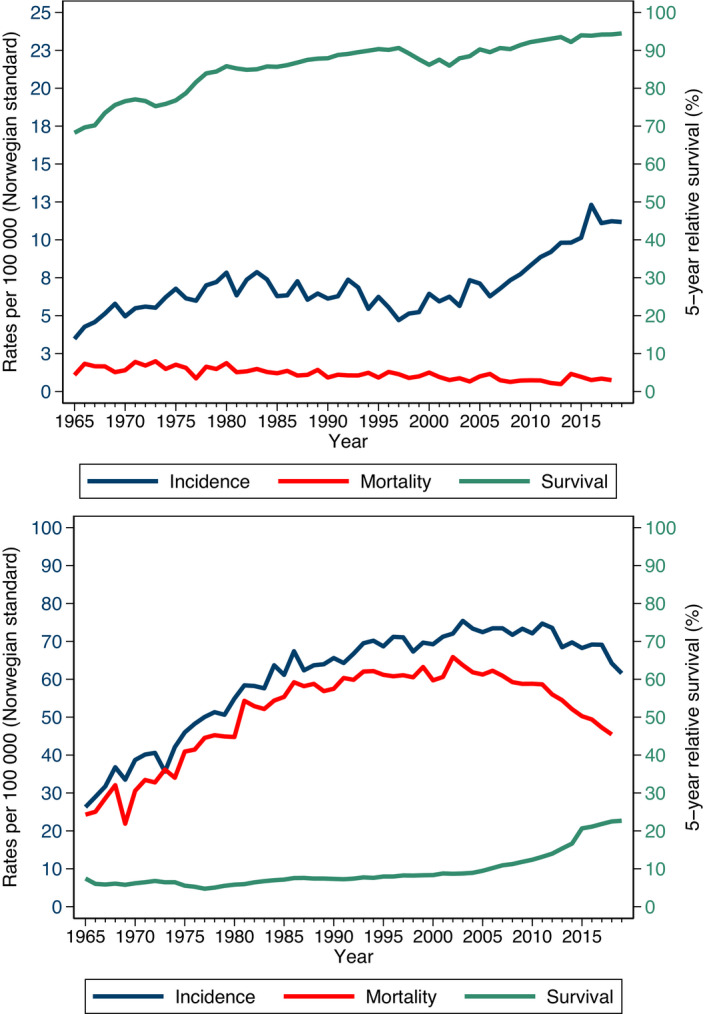
Incidence and mortality rates and 5‐year relative survival. (A) Incidence rates, mortality rates, and 5‐year relative survival for thyroid cancer among Norwegian women from 1965 to 2019. (B) Incidence rates, mortality rates, and 5‐year relative survival for lung cancer for Norwegian men from 1967 to 2019.

For illustration, incidence, mortality, and 5‐year relative survival for thyroid and lung cancer are shown in Fig. 2. The incidence of thyroid cancer among women has doubled in the last 20 years, whereas mortality has decreased marginally, and survival has increased slightly from an already high level (Fig. 2A) [7]. More sensitive data and a more careful analysis would be required to elucidate the underlying causes of these trends, but there is reason to believe that the sharp increase in incidence is due in large part to greater use of advanced diagnostic techniques: ultrasound examination; computed tomography; magnetic resonance imaging scanning; and more sensitive biomarkers. That is, a larger number of relatively nonaggressive thyroid cancers, that would not have been detected in the past, are now being diagnosed and contributing to estimates of patient survival.

We see in Fig. 2B that lung cancer mortality among Norwegian males increased steadily from 1965 until 2000 and then started to decline [7]. Changes in diagnosis and treatment have had only a limited impact on lung cancer survival. The trends in mortality are primarily driven by changes in incidence, which are, in turn, driven by the success of smoking cessation programs. Survival, which measures the effectiveness of the healthcare system at diagnosing and treating those cancers that arise, remained at a relatively low level from 1965 until 2005, although some improvements have been seen in recent years [7]. These figures illustrate the importance of studying all three measures to assess the cancer burden.

Each of the transitions (incidence, mortality, survival) can be expressed as either the rate at which it occurs (also called the intensity or hazard) or the probability at which it occurs. The two are mathematically related, and we may present either (or both). It is, however, more common to present incidence and mortality as rates (i.e., using person‐time as the denominator) and survival as a probability/proportion (using number of individuals as the denominator). We note that ‘mortality’ is the cancer mortality rate among the entire population, whereas survival can also be thought of as (a transformation of) the cancer mortality rate among only those individuals with a cancer diagnosis. Although incidence is commonly reported as a rate, it is sometimes also reported as a proportion/probability. For example, we may see reports of the ‘lifetime risk of being diagnosed with breast cancer’ (where the denominator is the number of individuals alive at a certain age or time)—the quantity epidemiologists call the cumulative incidence or incidence proportion and where ‘lifetime’ may be defined as, for example, ‘up to age 75’.

There are complex time dynamics underlying each of these measures that need to be understood to fully interpret them. Incidence rates, for example, vary as a function of both age and calendar time. Underlying these is a latent time scale, the natural history of cancer, which is not measured and varies markedly between tumors and individuals. The date of diagnosis of cancer is assumed to be well defined for the purpose of analysis, but diagnosis of cancer is a process rather than a fixed point in time, and the process occurs at different points in the natural history for each individual. The incidence during any given year will therefore depend on the distribution of time from onset to diagnosis for that particular year, which will depend on factors such as tumor biology, screening intensity, and diagnostic accuracy.

It is important to recognize that, whenever a probability is presented, it is for a well‐defined segment of the relevant time scale; the probability of developing cancer will be for a specified age range and the probability of surviving cancer will be ‘survival up to a specified time following diagnosis’.

### 2.2 Competing risks

**Fig. 3:**
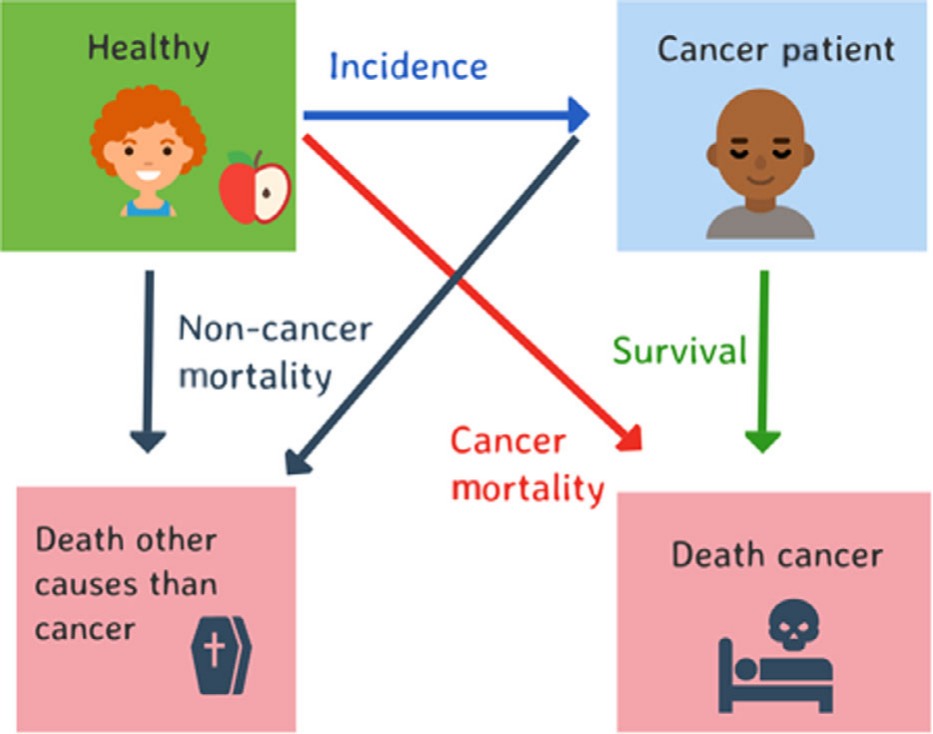
Conceptual overview of the measures that describe the rate of transitioning from one health state to another, competing risks are included.

In Fig. 3, we recognize that patients may also die of causes other than cancer. This introduces what is referred to as ‘competing risks’. In the transition from ‘healthy’ to ‘cancer patient’, for example, death due to other causes may occur before a cancer diagnosis. Death ‘competes’ with cancer to be the event that occurs first. When estimating the proportion of individuals who will develop cancer, we must be clear to define the age range. For example, we might be interested in the proportion of women who will be diagnosed with breast cancer before age 75. In a competing risk scenario, we can estimate two different types of probabilities: the real‐world probability and the hypothetical world probability. We can choose to estimate either the real‐world proportion of women who will be diagnosed with breast cancer before age 75 in the presence of competing risks of death (where death due to other causes precludes a diagnosis of breast cancer), or we can estimate the hypothetical proportion of women who will be diagnosed with breast cancer in the scenario where women cannot die of other causes before age 75. The real‐world estimate is more relevant for planning health services and cancer control activities, but the hypothetical estimate may be more relevant for comparing different populations because comparisons will be independent of noncancer mortality. We will elaborate on these concepts when discussing measures of survival, where both the hypothetical world (called net survival) and the real‐world (called crude survival) probabilities are commonly used. Our main point is that both measures are useful, but for different purposes. Hypothetical world measures are often most appropriate for evaluating scientific hypotheses, whereas planning of health services is best achieved using real‐world measures. It is important to recognize that a range of measures exist and to choose the measure most appropriate for the task at hand.

### 2.3 Risk, incidence rate, and standardization

#### 2.3.1 Risk

In lay terms, risk is ‘the possibility of something bad happening’ [8]. In epidemiology, risk is often defined as ‘the probability of an event during a specified period of time’, but is more correctly the probability as a function of time [9]. The key point is that a time period must always be specified when reporting risk(s). In the simplest scenario, with only one outcome (i.e., no competing events) and the ability to potentially follow all individuals for the complete period of time (no censoring), risk can be calculated as the proportion of individuals who experience the outcome of interest during a specified period of time. The calculation, assumptions, and interpretation become more nuanced when censoring and/or competing risks are present [9]. In the presence of competing risks, ‘risk’ is defined as the ‘real world’ (crude) probability, rather than the net probability, and is also called the incidence proportion or cumulative incidence.

#### 2.3.2 Incidence

The term ‘cancer incidence’ may refer to the number of incident cases, the incidence rate, or the incidence proportion; the precise measure being reported is usually clear in context. Measuring incidence rate diminishes the problem of competing risks (Table 1). The numerator is still, as in estimates of risk, the number of cases that occur in a defined population during a specific period, but the denominator is now the person‐time (rather than number of individuals as in risk) in the population. Hence, individuals contribute person‐years to the denominator only as long as they are alive during follow‐up.

**Table 1:** Overview of different measures of cancer incidence.

Measure	Definition	Pros and cons
Risk or incidence proportion or cumulative incidence	The probability of an event during a specified period of time (or as a function of time)	Pros: Easy to understand Cons: When competing risks are present, they also affect the risk
Crude incidence rate	Number of individuals who develop the cancer divided by total time at risk (person‐time) experienced by the individuals followed	Pros: Takes competing risks into consideration Cons: Subject to confounding when compared between populations with different age (or sex) distributions
Standardized incidence rate	Number of individuals who develop the cancer divided by total number at risk with each stratum (defined by age and sex) assigned weight from a defined external (hypothetical) population	Pros: Takes competing risk into consideration Allows unconfounded comparison with populations with a different age and/or sex distribution Con: Is hypothetical and will differ for any specific population depending on the standard population

The crude incidence rate is the simplest summary measure generated by dividing the total number of cases with the total person‐time in the population of interest. But the crude rate lacks generalizability because any comparison with another population in which the distribution of age and/or sex differs will be confounded and therefore potentially misleading.

Because incidence rates of cancer subtypes vary by age and the majority vary by sex [10], no single incidence rate can convey nuanced information about the entire burden of any specific cancer in a population. To accommodate this complexity, incidence rates need to be calculated separately by age and sex. In many instances, however, this generates an abundance of information that is impractical, for example, for monitoring cancer incidence and mortality trends or for comparisons between populations.

#### 2.3.3 Standardization

Standardized incidence rates are used to improve the comparability of incidence rates between populations with, for example, different age/sex structures. Incidence rates are first calculated within each population for each stratum defined by age and sex. The age‐standardized incidence rate is then calculated as the weighted average of the stratum‐specific rates, where the same weights are used for every population. Weights are typically defined by the proportion of individuals belonging to that stratum in the chosen standard population.

As a corollary, the age‐standardized rate is hypothetical and depends on the choice of the standard population. Consider, as an extreme example, that cancer incidence rates from a European population are standardized to a population in a low‐resource country, heavily dominated by young individuals with only a small proportion above the age 50 or 60 years. Heavy weight would then be assigned to strata at the lower age range with the lowest age‐specific incidence in a European country and low weight to high ages when the overwhelming majority of cancers are diagnosed in a population with the demographic structure typical for European populations. Therefore, rates that are age‐standardized to the European standard population are used for our purposes.

The choice of standard population is to some extent a trade‐off between comparability and interpretability. We recommend choosing a standard population best suited for the specific research question. For example, if one is interested in comparisons with other countries, then it might be wise to use the so‐called Segi–Doll world standard, which is used in all volumes of Cancer Incidence in Five Continents [11]. If one is primarily interested in temporal trends within a country, then the population structure within the country during the most recent period is most informative as the standard population. Reporting crude and age‐adjusted rates according to both European standard population and Segi–Doll world standard population, alongside each other, may provide a more comprehensive picture.

### 2.4 Incidence rate

At first glance, incidence (as the number of cancers) might be considered the most informative metric to measure the burden of cancer and the resources needed for diagnosis, treatment, and follow‐up in a population. Cancer incidence—overall or by specific cancer sites and types—also conveys information about the total burden of carcinogenic exposures, whereas temporal trends enlighten us about progress (or lack thereof) in cancer prevention. However, two reservations need serious consideration before we embrace age‐standardized incidence rates as a readily available metric allowing valid comparisons between time periods and different populations.

Firstly, collecting high‐quality cancer incidence data requires a sophisticated infrastructure. Although deaths are registered in virtually all European populations by date and underlying cause, with varying degrees of accuracy, only 46% of the European population is covered by cancer registries. Beside accurate and continuously updated information on the population denominator by age and sex, high‐quality cancer registry data also require: (a) acceptance by the population—with legal support—that the benefit of cancer registration outweighs potential concerns that personal autonomy is violated; (b) timely, complete, and mandatory reporting of newly diagnosed cancer cases from both public and private in‐ and outpatient care; (c) resources for proper histopathologic diagnosis whenever tumor tissue or cells are available for examination; and (d) central facilities for computerization, quality checks, regular processing and publishing of cancer incidence data, and secure long‐term storage. These challenging prerequisites are currently met in only a limited number of European countries.

Secondly, the recorded cancer incidence can be distorted. Screening for early detection of cancer can convey benefit only if diagnosis is made earlier, when cure might be more achievable. This advancement of diagnosis, the lead time, identifies some cancers which, in the absence of screening, would have surfaced clinically later, usually within a few years. Under this scenario, introduction of screening—whether organized or opportunistic—would create an initial increase in incidence followed by a decline. More recently, overdiagnosis of nonlethal cancer has become apparent as a consequence of novel sensitive diagnostic technologies, increased diagnostic activity, and particularly widespread cancer screening. This phenomenon is particularly well documented and quantitatively substantial for cancers of the prostate, breast, thyroid, kidney, and malignant melanoma [12]. Changes in classification and completeness of registration can further distort temporal trends in recorded cancer incidence.

Unfortunately, no methodology exists that can adjust for the impact of lead time bias or overdiagnosis on the recorded incidence of cancer. Over time, this problem is more likely to increase than to decline as new sensitive diagnostic technologies become available. As a corollary, trends in cancer incidence need cautious interpretation with proper consideration of the clinical context. For some cancers, particularly prostate cancer following widespread prostate‐specific antigen screening, trends in incidence rate have become almost uninterpretable if the purpose is to understand whether the occurrence of clinically significant disease is decreasing, stable, or increasing.

### 2.5 Mortality rate

Mortality and survival are often used as antonyms without any concern that the measures are defined differently in different fields and specialties in health care. When working with population‐based measures for cancer, mortality is the rate among the entire population and survival is the probability of survival calculated only among the cancer patients. The complement of survival (i.e., one minus survival) is lethality, not mortality, but is also known as the case fatality rate (despite being a proportion rather than a rate). Different measures of population mortality are used (Tables 2 and 3). Usually, mortality is standardized to a defined age and sex structure and shown as number of deaths per 100 000 person‐years. A database or registry of the population, all deaths, and the date of death, and an accurately coded underlying cause of death, is required to calculate annual mortality rates by cause of death. A further complication arises in that the recording and reporting of mortality statistics requires each death to be classified as having one underlying cause and potentially several contributing causes, but it may be difficult or even impossible to distinguish between these in real life.

**Table 2:** Overview of different measures of cancer patient survival. As previously mentioned, the mortality rate (hazard function) and the survival function are mathematically related so we can present outcomes on a number of scales (mortality rate, probability of dying, probability of surviving). For ‘survival’, it is traditional to present ‘survival probabilities’. For crude probabilities, we prefer to present the crude probability of dying of cancer. The crude probability of surviving cancer (one minus the crude probability of dying of cancer) is the probability of either dying of a cause other than cancer or still being alive.

Measure	Definition	Assumptions, pros, and cons
All‐cause survival	Probability of surviving beyond a given time	Assumptions: Can identify date of death for all deaths Pros: No strong assumptions. Easy to calculate and interpret Con: Influenced by noncancer deaths
Net survival	Probability of surviving beyond a given time in the hypothetical scenario where cancer is the only possible cause of death. This is the target measure of ‘cause‐specific survival’ and ‘relative survival’	Assumptions: Conditional independence of death due to cancer and death due to other causes. Accurate classification of cause of death (cause‐specific framework) or appropriate population life tables (relative survival framework) Pros: Independent of mortality due to causes other than cancer, so is ideal for comparing survival between different populations or over time within the same population Cons: Complicated definition. Hypothetical scenario is not optimal in clinical setting
Net probability of death	Probability of dying of cancer before a given time in the hypothetical scenario where cancer is the only possible cause of death. Calculated as 1 minus net survival	Same as for net survival
Crude probability of death	Probability of dying of cancer before a given time in the in the presence of other causes of death (i.e., in the real world). Epidemiologists know this as the ‘cumulative incidence of death’, but the terms ‘crude’ and ‘net’ are standard in the cancer survival literature	Assumptions: Accurate classification of cause of death (cause‐specific framework) or appropriate population life tables (relative survival framework). Do not require conditional independence assumption Pro: Directly relevant for patients and clinicians Con: Influenced by noncancer deaths
Proportion cured	Proportion of patients with a cancer diagnosis who are cured (will not experience excess mortality)	Assumptions: Same as for net survival plus the assumption that cure is reached (patients do not experience any excess mortality after some cure point) Pros: Easy to interpret. A single measure rather than a function of time Cons: Very sensitive to the assumption that cure is reached. Cannot be estimated if cure is not reached
Life expectancy	Number (or proportion) of life years lost due to a diagnosis of cancer Or Number of life years gained due to prevention or intervention in cancer care	Assumptions: Requires extrapolation to the point in follow‐up where all patients have died. Often life year lost (or gained) is estimated based on cancer‐specific death, not overall death. This may not be appropriate as even though there is a loss or gain in life years due to cancer‐specific cause of death, there may be no loss or gained life years overall Pros: Easy to interpret. A single measure rather than a function of time Con: Requires extrapolation
Conditional estimation	The abovementioned measures are typically estimated from diagnosis. All measures can be estimated conditional on having survived some initial period	Pros: Directly relevant for patients who have survived an initial period. Mortality is often high in the first year so providing estimates of 1‐year survival along with 5‐year survival conditional on one year can give a better summary than just 5‐year survival

**Table 3:** Overview of different measures of mortality

Measure	Definition	Pros and cons
All‐cause mortality^a^	Mortality from all causes	Pros: No misclassification of cause of death. Require no assumptions. Not related to time of diagnosis Cons: Cancer (specific) death may be a rare event and prevention or interventions on cancer may not influence all‐cause mortality
Total cancer mortality^a^	Mortality from all cancers combined	Pro: Less misclassification of cause of death Con: Prevention or interventions on a specific cancer may not influence total cancer mortality
Cancer‐specific mortality^a^	Mortality from a defined cancer site or type; sometimes from a specific site defined anatomically or by histopathology	Pro: Prevention and interventions are targeted to a specific cancer Con: Misclassification of cause of death
Incidence‐based mortality^a^	Cancer‐specific mortality counting only deaths after cancer diagnosis within a defined period	Pro: Allows for including only deaths from patients diagnosed after prevention or intervention on cancer Con: Misclassification of age of diagnosis if diagnosis is influenced by early diagnosis (lead time)

^a^ The entire population is the denominator and to facilitate comparison, results are usually standardized to a defined age structure and shown as number of events per 10^5^ person‐years.

A sometimes‐neglected bias arises when mortality is analyzed in cohorts of individuals with no history of cancer at the beginning of follow‐up. Hence, deaths from the cancer of interest occur only among those diagnosed after enrollment at time zero. In contrast, mortality rates in the general population are generated by cancers diagnosed during a much longer period without any left truncation. Application of these population rates—including death from cancers that are prevalent at time zero—will therefore exaggerate the expected number of deaths in the cohort, particularly during the early period of follow‐up. As a corollary, standardized mortality ratios in the analytic cohort will be underestimated. Such bias can be eliminated by use of incidence‐based mortality, which implies that the expected mortality is based only on deaths from cancers diagnosed after a defined date, typically when follow‐up of the cohort began (Table 3).

### 2.6 Survival

#### 2.6.1 Overview of alternatives

An exact mathematical relation exists between the survival proportion in a population and the mortality rate in that same population (equation 1 in [13]). One might think, therefore, that ‘mortality’ and ‘survival’ are just mathematical transformations of the same underlying concept. In cancer epidemiology, however, ‘mortality’ refers to the mortality rate in the population (not restricted to cancer patients), whereas ‘survival’ refers to the proportion surviving among a cohort of patients diagnosed with cancer. That is, ‘mortality’ and ‘survival’ use different denominators.

Although, at first sight, it might seem that estimates of cancer patient survival (the proportion of patients who survive a given period subsequent to diagnosis) are easy to calculate and communicate, this is not the case. Considerable nuances exist in how such measures can be interpreted and the contexts in which they are relevant [14]. The first challenge is that cancer patients may die of either cancer or other, nonrelated, causes (Fig. 3). We can choose to estimate any, or all, of the following three measures of survival:

All‐cause survival: the probability of surviving beyond a given time (e.g., 5 years) without dying of any specific cause.
Net survival: the probability of surviving beyond a given time (e.g., 5 years) without dying of the specific cancer of interest in the hypothetical scenario where the cancer of interest is the only possible cause of death because competing risks are assumed to be eliminated.
Crude survival: the probability of surviving beyond a given time (e.g., 5 years) without dying of the specific cancer of interest in the real‐world scenario where it is possible to die from other causes of death. That is, cause‐specific survival in the presence of competing risks.

Hence, net survival is a hypothetical world probability and crude survival is a real‐world probability. The term ‘crude’ is sometimes used to mean ‘unadjusted’; this is not what we mean here by crude survival. The terms crude and net come from the theory of competing risks; it is unfortunate that crude is used in another context but net survival and crude survival, as defined above, are standard terms when studying the survival of cancer patients [14, 15].

Our focus is on population‐based measures and not on surrogate endpoints, such as progression‐free survival, that may be of interest in clinical trials. Recent tutorial papers provide overviews of survival measures (including some not discussed here) that are of potential interest in a clinical setting, such as conditional survival, the cure proportion, and loss in expectation of life (Table 2) [14, 15].

#### 2.6.2 All‐cause survival

All‐cause survival is relatively easy to calculate and interpret using routine data because one does not have to consider the cause of death. For cancer outcomes research, however, we are typically interested in estimating the probability that patients will die of their specific cancer (i.e., net or crude survival). All‐cause survival captures all deaths, including deaths from causes other than cancer, so temporal trends will include concomitant trends in mortality from nonmalignant diseases. Because noncancer mortality has been declining in many populations, all‐cause survival of cancer patients will increase even without any improvements in cancer‐specific fatality. All‐cause survival also has advantages under certain circumstances because it is independent of criteria for classification of cause of death, which may differ between settings, time periods, or groups being compared.

#### 2.6.3 Measures and frameworks

After establishing that our interest lies in cancer‐specific death, rather than all‐cause death, as the outcome, it is important to distinguish between measures (net and crude probabilities), the framework (cause‐specific or relative) for estimating the chosen measure, and the choice of estimators within the chosen framework [16]. The first step is to choose a measure. That is, whether one wishes to estimate net survival (survival in a hypothetical world where competing risks are eliminated) or crude survival (survival in the presence of competing risks). Each of these measures can be estimated within either a cause‐specific framework (where we require accurate information on cause of death) or a relative survival framework (where we use tabulated population mortality rates to account for noncancer mortality). Table 4 provides an overview of the hierarchy and examples of some of the estimators. We will first discuss measures before moving to a discussion of the frameworks in which these measures can be estimated, such as relative survival versus cause‐specific survival.

**Table 4:** Overview of the two frameworks and measures of cancer patient survival.

	Measure	
	Net survival: competing risks eliminated	Crude survival: in the presence of competing risks
Framework		
Cause‐specific: use cause of death information to identify cancer deaths	Cause‐specific survival: Censor survival times of noncancer deaths and apply standard estimators such as Kaplan–Meier	Crude probability of death using cause of death: Standard estimators of the cumulative incidence function in the presence of competing risks
Relative survival: contrast all‐cause survival of cancer patients to survival of the general population	Net survival: Can be estimated using age‐standardized relative survival (Ederer II) or the Pohar Perme estimator of net survival	Crude probability of death in a relative survival framework: Life table approach (Cronin & Feuer) Model‐based approach

##### Measures

###### Net survival

As mentioned above, net survival aims at estimating the prognosis of a certain cancer in the absence of other causes of death. When estimating cancer patient survival using data collected by population‐based cancer registries, net survival is the most popular measure. Hence, most published estimates of cancer patient survival lack a real‐world interpretation as they assume that deaths due to causes other than cancer do not exist [14]. While this assumption may seem odd at first glance, it is precisely the quantity of greatest relevance when comparing survival among groups with different noncancer mortality rates.

Assume that our interest is in comparing the survival of patients diagnosed with colon cancer across countries with large demographic differences in income level and education. Because such differences are known to be associated with life expectancy, incorporating noncancer mortality in the estimation would yield unfair comparisons of cancer survival. To isolate the effect of cancer on the risk of dying in each country, the probability of dying is estimated under the assumption that it is not possible to die of causes other than cancer.

The International Cancer Benchmarking Partnership [17], CONCORD [18], and EUROCARE [19] projects are examples of ongoing international collaborations that report net survival. Their aim is to provide an evidence base for global cancer control and international comparisons of the effectiveness of healthcare systems as a basis for policy and practice changes toward reducing the burden of cancer. Net survival is also the measure of choice when reporting estimates of patient survival within a single country; net survival is independent of noncancer mortality, whereas crude survival is affected by noncancer mortality.

###### Crude survival

Net survival is the most commonly reported measure of population‐based cancer patient survival, but the hypothetical framework is not optimal for healthcare professionals and patients who live in the real world. Rather than presenting crude survival (as defined above), it is more common to present the crude probability of death due to cancer (1 minus crude survival). This is because ‘dying of cancer’ is relatively well defined, whereas ‘not dying of cancer’, that is, survival, encompasses both being alive and dying of other causes. The crude probability of death due to cancer is also known as the cumulative incidence of death due to cancer, but the terms crude and net are well established in the cancer patient survival literature.

The crude probability of cancer death is the probability of dying of the cancer of interest before a specified time where it is possible to die from competing causes of death. The crude probability of death is perhaps more relevant in a clinical setting but is affected by both cancer and noncancer mortality hazards and therefore less relevant for comparisons between different groups or populations. Even if two groups of cancer patients have a similar cancer hazard, the group with a higher noncancer hazard will have a lower crude probability of death due to cancer because death due to other causes will ‘prevent’ them dying of cancer.

###### Trade‐off between comparability and interpretability when choosing a survival measure

Net survival, which is invariant to changes in noncancer mortality, is constructed to facilitate comparison of survival between populations (e.g., between countries or over time in a single population). Because this results in a ‘hypothetical world’ interpretation, it is not an optimal measure for predicting the prognosis of actual patients. Net survival is usually age‐standardized, in the same manner as we standardize incidence and mortality. We may read, for example, that the estimated age‐standardized 10‐year net survival for patients with a given cancer in a given country is 75%. This estimate was obtained in order to facilitate comparisons with other populations. We should not try to interpret it as a proportion of real‐life cancer patients who survive for longer than 10 years. It is the proportion of cancer patients who survived longer than 10 years in the hypothetical scenario where cancer was the only cause of death, and the age distribution of patients with cancer was that of the standard population rather than the actual age distribution. If one is interested in measuring the proportion of patients who survive 10 years in the real world, crude survival or all‐cause survival are preferable, and estimates should be made for separate, narrow age ranges rather than age standardization.

##### 2.6.3.1 Frameworks

Two different frameworks exist to estimate net and crude survival, namely cause‐specific and relative survival (Table 4).

###### Cause‐specific survival

In the cause‐specific framework, each death must be classified as being either completely due to the cancer of interest or completely due to other causes. Such a classification may be difficult to make by the treating physician, who has complete information; it is even more difficult to make based on routinely recorded information on death certificates, cause of death registers, or medical records, which is the norm for population‐based studies. In the statistical analysis, cancer deaths are denoted as events and deaths due to other causes are treated as censored observations. Standard methods (e.g., Kaplan–Meier) are applied to estimate what is often called cause‐specific survival. It is not always appreciated that these are estimates of net survival (under certain assumptions) and have the hypothetical world (of disregarding other causes of death) interpretation. By censoring the survival times of patients who die of causes other than the specific cancer, we are eliminating the possibility of dying of these causes. Patients who die of causes other than the specific cancer are treated in the analysis in the exact same way (their survival time is censored) as those who are alive at the end of follow‐up.

###### Relative survival

The relative survival framework is an alternative for estimating net survival that does not require any, let alone accurate, information on cause of death. Relative survival is defined as the all‐cause survival of the cancer patients divided by the expected survival in a comparable group without the specific cancer. In practice, expected survival is estimated based on the mortality in the general population. If, for example, the one‐year relative survival ratio (1‐year survival among cancer patients divided by 1‐year survival in the general population) is 1.0, then all‐cause survival of the group of cancer patients coincides with that in the general population. This suggests that there is no excess mortality associated with a cancer diagnosis during the first year of follow‐up. A relative survival ratio lower than 1.0 means that cancer patients have inferior survival compared to the general population, and we typically assume that the survival deficit is due to excess mortality from the cancer.

Excess mortality captures both the direct and indirect mortality associated with the cancer. The mortality that is directly related to the cancer includes all causes of death that would typically be classified as death due to cancer on the death certificate. Indirect mortality due to cancer encompasses death due to, for example, treatment toxicity, suicides, and late effects of treatment such as cardiovascular disease, second malignancies, or infections. These events are unlikely to be classified as cancer deaths on the death certificate and are therefore not captured in a cause‐specific analysis. As cause of death information is not always readily available or reliable, relative survival has become the preferred approach to estimate cancer patient survival in population‐based investigations, as well as by cancer registries worldwide.

###### Pros and cons with cause‐specific versus relative survival

Each of the two frameworks for estimating net survival requires specific assumptions. The cause‐specific framework requires accurate classification of cause of death, whereas the relative survival framework requires appropriate estimation of the expected survival in the absence of cancer. National life tables are frequently used to estimate expected survival. This requires two assumptions: (a) that mortality due to the specific cancer constitutes a negligible proportion of total mortality since we are using the entire population to represent a population without the specific cancer; and (b) that the cancer patients would experience the same mortality as the general population if they were not diagnosed with cancer. In most scenarios, the use of national life tables results in a negligible bias, although it can result in a bias in net survival for common cancers among the elderly, and an even larger bias in net survival for all cancers combined because they account for a substantial proportion of all deaths in the general population [20].

For smoking‐related cancers, using national life tables may not be appropriate. If, for example, individuals with lung cancer, predominantly caused by smoking, had not developed this malignancy, we would expect them to have higher mortality than the general population because smoking increases the risk of numerous lethal diseases, including several other sites of cancer as well as cardiovascular and pulmonary diseases. We can partially correct for this bias by stratifying the general population life tables by, for example, socioeconomic status, as smoking is highly associated with socioeconomic status in addition to the usual age, sex, and calendar year [21]. Few cancer registries, however, record such data.

One study showed that, despite the concerns with using relative survival for lung cancer, the resulting bias was negligible due to the high fatality of lung cancer [22] and another found no evidence of a bias due to ignoring the effect of smoking for bladder cancer [23].

Although smoking serves as an obvious example of how the assumptions are violated, using the relative survival framework is most problematic for early‐stage screen‐detected cancers. For example, white males aged 65 years and older, diagnosed with localized/regional prostate cancer in the United States, had relative survival of 104% and cause‐specific survival of 95% [24]. The corresponding figures for black males were 101% and 93%. The men with prostate cancer experienced better survival than the age–sex–year–race‐matched general population because they were presumably more health conscious (and healthier) than the corresponding general population. Cause‐specific survival, on the other hand, is likely a slight underestimate of net survival because there is evidence from many populations that cancer is over‐reported as a cause of death among elderly individuals with a cancer diagnosis [25].

In summary, for population‐based estimates of cancer survival, the relative survival framework is generally preferred over cause‐specific survival, although the choice of framework should depend on an assessment of the assumptions for the specific data and research question at hand [25, 26, 27] (Box 1).

Box 1 **A technical note on relative survival**

There is a subtle, but important, distinction between the relative survival framework for estimation and relative survival estimated as the ratio of observed to expected survival (described above), which is an estimator within that framework.

Until recently, relative survival was thought to be an estimator of net survival with no need to distinguish between frameworks and estimators because they were thought to be one and the same. In 2012, Perme *et al*. [16] published a seminal paper showing that relative survival is a biased estimator of net survival. A new unbiased estimator—known as the Pohar Parmar estimator—is now becoming the estimator of choice. This estimator uses a relative survival framework but does not involve taking the ratio of observed to expected survival; that is, it uses the relative survival framework to estimate net survival but is not relative survival.

Both methods estimate net survival by contrasting the all‐cause survival of the patients to the expected survival based on general‐population life tables, but they are not both ‘relative survival’.

#### 2.6.4 Survival for all cancers combined

Net survival for all cancers combined is often presented with the aim of providing a single summary measure. The measure does not have a ready interpretation because cancer is a collection of many distinct disease entities. Interpreting temporal trends has limitations.

Assume a temporal decline in the incidence of highly fatal cancers such as lung and stomach cancer, and a concomitant increase in malignancies with a favorable prognosis and in addition with a risk of overdiagnosis, of nonlethal breast, such as prostate and thyroid cancer [12]. Such a change in the mixture of different cancer sites will create a spurious increase in overall survival even in the absence of any real therapeutic progress. If one wishes to interpret trends in this measure, then it should be standardized by cancer site in addition to the usual standardization by age [28, 29]. That is, take the weighted average of the net survival for each subsite using the same weights for all periods. When using the relative survival framework for specific cancers, we rely on the assumption that mortality due to the specific cancer makes up a negligible proportion of the all‐cause mortality in the general population. This assumption does not hold when studying survival for all cancers, so estimates will be potentially biased [20].

We advise against estimating and reporting net survival for all cancers combined because it does not have a meaningful interpretation and, if reported, it is highly likely to be misinterpreted. We recognize, however, that there is demand for a summary measure of cancer patient survival to monitor survival in a population, and net survival for all cancers is commonly reported. If it is to be reported then it must be standardized by age and site at a minimum, as described in Verdecchia *et al*. [29]. It should be called a survival index [28], and it should be stressed that the measure is only useful for comparisons and cannot be interpreted as a survival proportion.

#### 2.6.5 Interpretation of temporal trends

In the context of the Academy initiative, improved 10‐year prognosis has been defined as a central measure of success. Proper interpretation of temporal trends in survival is, however, never straightforward [30]; it requires a deep understanding of the pros and cons with the particular measure. It requires understanding of the clinical context and changes in diagnostic intensity and management. Ideally, it also requires access to data on incidence and mortality as well as an attempt to estimate the potential impact of lead time bias and overdiagnosis bias. Because the goal has been defined for overall 10‐year cancer‐specific survival, adjustments may also be necessary for the confounding effect of changes over time in the distribution of various cancer sites and types. The complexity of this undertaking should not be underestimated. In order to obtain a valid overall estimate, the process must begin with a detailed assessment of each cancer site and type.

### 2.7 Health‐related quality of life

#### 2.7.1 Overview

The COVID‐19 experience has recently reminded us that the risk of dying is not the only perspective most individuals consider important. During the pandemic, many European countries isolated elderly people in a restrictive manner in order to reduce their risk of infection and protect their life. Besides all the advantages of these measures and their benefits, as measured by mortality, some elderly people clearly stated that they would have preferred to risk their life rather than suffer from isolation and develop mental health problems [31, 32]. This extreme situation emphasizes that a broader multidimensional perspective is needed that covers quality of life aspects, also among cancer patients.

To fully understand the complex cancer burden, we need to capture the patients' experience of their disease, treatment, and often lifelong surveillance by measuring quality of life. This need is increasingly embraced also in the context of clinical trials and by regulatory authorities. Hence, we must foster more patient‐centered research that incorporates all stakeholders' perspective [33]. Quality of life and patient‐reported outcomes have also been reported to predict survival in different types of cancer, although the mechanisms still need to be identified [34, 35, 36, 37]. The World Health Organization defines quality of life as an individual's perception of their position in life in the context of the culture and value systems in which they live, and also in relation to their goals, expectations, standards, and concerns.

#### 2.7.2 Generic quality of life questionnaires

The multidimensionality of quality of life requires assessment of physical, functional, emotional, and social well‐being, satisfaction, and relationships, parts of which are subjective. Health‐related quality of life, as distinguished from quality of life, is more specific and appropriate in clinical research and practice, because it focuses on aspects of life that are affected by disease and healthcare interventions. The currently most common way of measuring quality of life is by the use of written or online questionnaires filled in by the patients. Repeated use of self‐reported measures allows follow‐up of patients over time, with construction of trajectories of different functions and symptoms.

A large number of well‐constructed and psychometrically valid quality of life questionnaires have been developed (Table 5). Generic tools that measure broad aspects of physical, emotional, and social function are intended for general use, irrespective of the illness or condition of the patient. Commonly used generic questionnaires are the EQ‐5D [38] the Short Form‐36 Item Survey (SF‐36) [39] and the identical RAND‐36 Item Health Survey [40]. While RAND‐36 is a public domain form, SF‐36 is copyrighted and commercially distributed [41]. These tools, often applicable also to healthy people, provide an overall assessment of the respondent's quality of life. However, they fail to assess clinically important aspects of a cancer patients' health‐related quality of life. This limitation has stimulated development of disease‐specific questionnaires, which address the symptoms and psychosocial complaints that often occur among a subset of patients. Such questionnaires may be either highly specific or suitable for a larger group of diseases, including cancer.

**Table 5:** Overview of different measures of quality of life. EQ‐5D, EuroQol 5 Dimension; SF‐36/12, 36/12‐Item Short Form Survey.

Measure	Definition	Pros and cons
EQ‐5D	Generic quality of life questionnaire	Pros: Independent on health status. Compare different groups of people. Short and quick to respond to. Commonly used in health‐economic evaluations and QALY analyses Con: Lack clinically important aspects of a patients' health
SF‐36/12 RAND‐36	Generic quality of life questionnaire	Pro: Independent on health status. Compare different groups of people Cos: Lack clinically important aspects of a patients' health
EORTC QLQ‐C30/QLQ‐C15‐PAL	Cancer disease‐specific questionnaire with a palliative version	Pros: Functions and symptoms common among cancer patients in general. All cancer patients. Connected with site‐specific modules Con: Not possible to compare with other groups of people
EORTC QLQ‐XX	Cancer site‐specific modules to connect to the EORTC QLQ‐C30/ QLQ‐C15‐PAL	Pros: Symptoms and problems commonly occurring in the site‐specific cancer diagnosis. Modules for many different cancer diagnoses are available Cons: With core questionnaire together with a site‐specific module there will be 40–60 items to reply to. Not possible to compare with other groups of people
EORTC Aspect specific	Questionnaires measuring different aspects of cancer survivorship	Pros: Areas of survivorship are investigated in more detail. Many aspect specific questionnaires available (e.g., about cancer survivorship fatigue, cachexia, sexual health and satisfaction with care) Con: Does not give a broader picture of quality of life
FACT‐G/G7	Cancer disease‐specific questionnaire with a short version	Pros: Functions and symptoms common among cancer patients in general. All cancer patients. Connected with site‐specific modules Con: Not possible to compare with other groups of people
FACT‐X	Cancer site‐specific modules to connect to the	Pros: Symptoms and problems commonly occurring in the site‐specific cancer diagnosis. Modules for many different cancer diagnoses are available Cons: With core questionnaire together with a site‐specific module there will be 40–60 items to reply to. Not possible to compare with other groups of people
FACT Aspect specific	Questionnaires measuring different aspects of cancer survivorship	Pros: Areas of survivorship are investigated in more detail. Many aspect specific questionnaires available (e.g., about anemia, lymphedema and body image) Con: Does not give a broader picture of quality of life

#### 2.7.3 Cancer‐specific quality of life questionnaires

One of the two most widely used cancer‐specific questionnaires was developed by the European Organization for Research and Treatment of Cancer (EORTC): the EORTC Quality of Life Questionnaire‐Core 30 (EORTC QLQ‐C30) [42, 43]. The other is the Functional Assessment of Cancer Therapy‐General (FACT‐G) scale [44]. Both these questionnaires have short versions suitable for patients in palliative care. In addition to cancer site‐specific modules, questionnaires have been developed that measure symptoms and problems common for particular cancer sites. A comparison between EORTC QLQ‐C30 with FACT‐G revealed that they cover slightly different aspects of quality of life and that the subscales are not directly comparable [45]. Therefore, selection of a core patient‐reported outcomes measure should be based on the purpose of investigation.

In addition to the core cancer questionnaires with site‐specific modules, questionnaires measuring different aspects of cancer survivorship, such as fatigue, cachexia, anemia, and body image, are available from both EORTC and FACT, but the range may differ (Table 5). Currently, the EORTC Quality of Life group is developing a new core questionnaire focused on cancer survivorship to capture a bigger picture of the patient's situation. This questionnaire can be used as a stand‐alone or in combination with a cancer site‐specific (survivorship) module [46].

Of importance when measuring patient‐reported outcomes is to distinguish clinically relevant from statistically significant differences. Therefore, several questionnaire developers provide guidelines for levels of clinically relevant differences. The EORTC, for example, has developed such guidelines for their core questionnaire EORTC QLQ‐C30 [47, 48].

Due to the fact that information on quality of life can be achieved by self‐assessment, new technologies, such as smartphones, tablets, and wearables, open new opportunities to address health‐related quality of life. Assessments based on these technologies can be performed by the patients at any time and place. This facilitates longitudinal monitoring and may allow even more frequent and flexible assessments. Using static standard questionnaires, everyone answers the same questions. Hence, some questions may not be relevant or informative for all patients. If the provided information is directly analyzed, an individualized adaption of the follow‐up questions is tempting. The most prominent example is the EORTC QLQ CAT [49], a computerized test (CAT) where the questionnaire is adapted to the individual patient.

To capture a bigger picture of the patients' experience of their disease and treatment, a combination of several questionnaires may be needed. However, evaluation with several questionnaires may burden the patient and the selection of measurements therefore needs consideration. As of today, there is no standardized patient‐reported outcome measure that covers a full picture of the cancer patient's life situation with a limited number of questions. To develop such a measurement that takes advantage of modern communication technologies is an appealing challenge for the future.

In some instances, there are also opportunities to assess parts of the patient‐reported outcomes more objectively, via specific devices or other data sources, and further analyze the data via computer‐based algorithms. Although some aspects of health‐related quality of life, such as sleep quality, may sound easy to assess, they are complex constructs that cannot yet be captured via objective measures [50]. To measure sleep quality, it is necessary to distinguish between lying down and watching TV or lying down and sleeping, but accelerometers for sleep cannot yet assess this. Most probably, future assessment of health‐related quality of life and other patient‐reported outcomes will be based on a combined use of instruments that exploit the benefits of each option. To evaluate medical interventions or prevention programs in relation to health economy and quality of life of the patients, quality‐adjusted life year assessments, QALYs [51], are commonly used, which include both the quality and the duration of life.

## 3 Recommendations and future developments

A fundamental prerequisite for a realistic, valid, and timely assessment of whether the Academy goals have been achieved is the development of a detailed analysis plan. This plan should have a specific focus on cancer‐specific survival and mortality—the Academy's two overarching goals. However, time is ripe for a broader perspective of cancer outcomes research that also includes primary prevention and quality of life. Preparatory work that needs to be initiated before long also includes validation studies, harmonization of terminologies, and standardization of procedures to make comparisons between countries and time periods informative.

We recommend the use of incidence and mortality rates standardized to the European population. Assessing cancer patients' survival—and particularly temporal trends in prognosis—is more complicated and offers more methodologic alternatives than generally appreciated. As we have discussed in detail, none of these methods is ideal and they are based on data not readily available in all European countries. On balance, however, net survival is our recommended choice. When overall cancer net survival is estimated—as recommended by the Academy—special measures are required to adjust the expected rate of death in the general population and eliminate confounding due to temporal changes in the distribution of cancer sites.

Finally, we recommend use of cancer‐specific—rather than generic—instruments to assess quality of life; a combination of existing questionnaires to accommodate both features specific for different cancer sites and the patient's general condition; and dedicated efforts to use emerging communication technologies such as smartphones when possible. The latter approach might indeed allow quality of life assessment with a substantially deeper level of resolution than hitherto possible.

Similar to other branches of science, methodologies in outcomes research will undoubtedly undergo further development. To integrate measures of survival, quality of life and costs is indeed a formidable challenge, fundamentally important for rational use of constrained healthcare resources. Optimized use of novel communication technologies offers entirely new avenues for primary prevention, quality of life assessment, and evidence‐based surveillance of the ever‐increasing number of cancer survivors in Europe [52]. A growing number of randomized trials have a noninferiority design, which offers a new dimension to outcomes research [53]. We also foresee a new generation of randomized trials in which blinding is not feasible but long‐term follow‐up necessary. Hence, while confounding is eliminated at the time of randomization, it may arise during extended follow‐up and adjustment requires continued monitoring of such confounders [54].

We conclude that outcomes research is challenging, increasingly complex, and profoundly important. In Part II of this review, we will discuss the practical aspects of outcomes research in Europe—the opportunities and the sine qua non for a reliable assessment of progress.

## Conflict of interest

The authors declare no conflict of interest.
